# Proteomic analysis of the monkey hippocampus for elucidating ischemic resistance

**DOI:** 10.3164/jcbn.19-78

**Published:** 2020-04-09

**Authors:** Yurie Mori, Shinji Oikawa, Shota Kurimoto, Yuki Kitamura, Saeko Tada-Oikawa, Hatasu Kobayashi, Tetsumori Yamashima, Mariko Murata

**Affiliations:** 1 Department of Environmental and Molecular Medicine, Mie University Graduate School of Medicine, Edobashi 2-174, Tsu, Mie 514-8507, Japan; 2 College of Pharmacy, Kinjo Gakuin University, 2-1723 Omori, Moriyama-ku, Nagoya, Aichi 463-8521, Japan; 3 Department of Human Nutrition, School of Life Studies, Sugiyama Jogakuen University, 17-3 Hoshigaoka-motomachi, Chikusa-ku, Nagoya, Aichi 464-8662, Japan; 4 Departments of Psychiatry and Neurobiology, Kanazawa University Graduate School of Medical Science, Takakura-machi 13-1, Kanazawa, Ishikawa 920-8641, Japan

**Keywords:** hippocampus, dentate gyrus, oxidative stress, proteomics, carbonylation

## Abstract

It is well-known that the cornu *Ammonis* 1 (CA1) sector of hippocampus is vulnerable for the ischemic insult, whereas the dentate gyrus (DG) is resistant. Here, to elucidate its underlying mechanism, alternations of protein oxidation and expression of DG in the monkey hippocampus after ischemia-reperfusion by the proteomic analysis were studied by comparing CA1 data. Oxidative damage to proteins such as protein carbonylation interrupt the protein function. Carbonyl modification of molecular chaperone, heat shock 70 kDa protein 1 (Hsp70.1) was increased remarkably in CA1, but slightly in DG. In addition, expression levels of nicotinamide adenine dinucleotide (NAD)-dependent protein deacetylase sirtuin-2 (SIRT2) was significantly increased in DG after ischemia, but decreased in CA1. Accordingly, it is likely that SIRT2 upregulation and negligible changes of carbonylation of Hsp70.1 exert its neuroprotective effect in DG. On the contrary, carbonylation level of dihydropyrimidinase related protein 2 (DRP-2) and l-lactate dehydrogenase B chain (LDHB) were slightly increased in CA1 as shown previously, but remarkably increased in DG after ischemia. It is considered that DRP-2 and LDHB are specific targets of oxidative stress by ischemia insult and high carbonylation levels of DRP-2 may play an important role in modulating ischemic neuronal death.

## Introduction

Oxidative damage due to ischemia-reperfusion has been implicated as one of the leading causes for cell death in a number of diseases such as cardiovascular diseases and neurological disorders.^(1–4)^ The brain is particularly vulnerable to oxidative stress, because it consumes large amounts of oxygen compared to other organs.^(5)^ Oxidative stress refers to a cell’s state characterized by excessive production of reactive oxygen species (ROS). ROS, such as hydroxyl radicals (^•^OH), participate in damaging the cytoplasmic proteins, membrane lipids and DNA.^(6,7)^ Oxidative damage to proteins can disrupt active site of enzymes, affect the conformation of structural proteins, and interrupt the protein function.^(8,9)^ Protein carbonylation, the major and most common oxidative modification of proteins,^(10)^ has been extensively focused to monitor oxidative damage due to its irreversible and irreparable nature,^(11)^ and shown to affect the function and/or metabolic stability of the modified proteins.^(12)^ Thus, protein carbonyls are likely to play an important role in the pathophysiology of disorders associated with oxidative stress.

Previous studies demonstrated that hippocampus, crucial for learning and memory processes, is extremely vulnerable to oxidative stresses.^(13–19)^ Especially, the cornu *Ammonis* 1 (CA1) sector of hippocampus is known to be extremely vulnerable to the ischemic insult than the dentate gyrus (DG).^(20)^ Our previous study demonstrated carbonyl modification of heat shock 70 kDa protein 1 (Hsp70.1) in CA1 after the ischemia-reperfusion, and suggested that oxidative damage of Hsp70.1 may cause autophagy/lysosomal failure, leading to neuronal death.^(21)^

In this study, to clarify the mechanism of resistance to oxidative stress in DG, we investigated alternations of protein carbonylation and expression levels in DG of Japanese monkeys (*Macaca fuscata*) after the transient whole brain ischemia. The identification and characterization of carbonyl-modified proteins in the postischemic monkey DG were done by two-dimensional gel electrophoresis (2DE) with immunochemical detection of protein carbonyls (2D Oxyblot) and peptide mass fingerprinting (PMF). We also examined alternations of protein expression in DG after the ischemia-reperfusion, using two-dimensional differential in-gel electrophoretic analysis (2D DIGE). In addition, to elucidate the difference of vulnerability against oxidative stress between DG and CA1, we compared alteration of carbonyl levels in DG after the ischemia-reperfusion with those in CA1, which we previously reported.^(21)^

## Materials and Methods

### Sample preparation

All experimental procedures were performed in adherence with the guidelines of the Animal Care and Ethics Committee of Kanazawa University (Approval No.: AP-132874) and the NIH Guide for the Care and Use of Laboratory Animals. The monkeys used in this study were 12 adult (5–11 years of age) Japanese monkeys (*Macaca fuscata*) with body weight of 5–10 kg. We operated ischemia-reperfusion according to the procedure previously described.^(22,23)^ Briefly, after removal of the sternum, the innominate and left subclavian arteries were exposed in the mediastinum and were clipped for 20 min, then reperfusion was done. The effectiveness of clipping was demonstrated by an almost complete absence of cerebral blood flow, which was monitored by laser Doppler (Vasamedics, St. Paul, MN). Hippocampal DG and CA1 tissues were resected from both the sham-operated controls (*n* = 3), postischemic day 3 (*n* = 3), day 5 (*n* = 3) and day 7 (*n* = 3) monkeys at indicated time points after the ischemic insult under the general anaesthesia.^(23)^ The control monkeys were dissected on the same day of sham operation. Dissected fresh samples were immediately put into the liquid nitrogen and stored at –80°C until use.

Frozen tissue (20–30 mg) was directly transferred into a reaction tube containing 100 µl of lysis buffer (30 mM Tris-HCl, 7 M urea, 2 M thiourea, 4% w/v 3-[(3-cholamidopropyl) dimethylammonio] propanesulfonate (CHAPS), a protease inhibitor cocktail, pH 8.5). The tissue samples were homogenized using the Sample Grinding Kit (GE Healthcare UK Ltd., Buckinghamshire, England) and incubated for 60 min on ice. All samples were centrifuged at 30,000 × *g* for 30 min at 4°C. The supernatant was collected and stored at –80°C. Total protein of sample was quantified by the Bradford assay, using bovine serum albumin as standard.^(24)^

### Detection of carbonyl modified proteins (2D Oxyblot analysis)

The mixed protein samples (100 µg protein) were prepared by combining equal amounts (33 µg) of three different samples from each group (control, days 3, 5 and 7). Carbonylated proteins in the mixed protein samples (100 µg protein) were labeled by derivatization of carbonyl group with 2,4-dinitrophenylhydrazone (DNP) by reaction with 2,4-dinitrophenylhydrazine (DNPH) and separated by 2DE. Proteins were transferred onto the PVDF membranes (Immobilon-P Transfer Membrane, Millipore, Darmstadt, Germany), using TE77 semi-dry transfer unit (GE Healthcare, Buckinghamshire, England). Transferred membranes were blocked and incubated with anti-DNPH rabbit polyclonal antibody.^(21)^ The chemiluminescence signal was detected on X-ray films. The spot intensities of carbonylated proteins were quantified using PDQuest ver. 8.0 (Bio-Rad, California, CA). We repeatedly confirmed reproducibility of 2D Oxyblot analysis. The specific oxidation was estimated as relative carbonyl level (obtained from 2D Oxyblot) per relative protein expression (obtained from 2D DIGE). For the protein identification, spots were excised from 2D gels obtained with non-DNPH-treated samples and analyzed by the mass spectrometry.

### Two-dimensional differential in-gel electrophoretic (2D DIGE) analysis

Samples were labeled using CyDyes DIGE Fluor (minimal dye) labeling kit (GE Healthcare). Samples (25 µg protein) were labeled with minimal fluorescent dye and incubated on ice for 30 min in the dark. Then, each Cy3 labeled samples were combined with an equal amount of Cy5 labeled samples, and the pooled Cy2 labeled sample was added as an internal standard and mixed each batch. First dimension, isoelectric focusing (IEF), was performed on IPG strips (IPG, pH 3–10 NL strips, 24 cm, GE Healthcare) and an Ettan IPGphor isoelectric focusing system (GE Healthcare). Samples labeled with fluorescent dye were applied strips on the reswelling tray and focused. After focusing, proteins were separated by 2DE.^(21)^ The 2D DIGE gels were scanned in a Typhoon FLA 9500 (GE Healthcare). Spot detection, gel matching, and statistical analysis were performed with DeCyder 2D software ver. 7.2 (GE Healthcare). Protein expression values were statistically analyzed using Student’s *t* test.

### Protein identification

The method used was essentially as described by Kondo *et al.*^(25)^ with some modifications. Preparative 2DE gels were visualized by CBB-stained. The protein spots picking were performed manually, were destained four times, dehydrated twice, and digested with trypsin (Promega, Madison, WI) solution for overnight at 37°C. The peptide solutions were eluted with 45% acetonitrile/0.1% trifluoroacetic acid (TFA) and concentrated. The peptide solutions containing 50% α-cyano-4-hydroxycinnamic acid (Wako Pure Chemical, Osaka, Japan) were spotted on a MALDI plate. Mass analysis was performed with a matrix-assisted laser desorption ionization time-of-flight tandem mass spectrometry (MALDI-TOF/TOF MS; 4800 *Plus* MALDI-TOF/TOF^TM^ Analyzer, AB SCIEX, Framingham, MA). Protein identification was performed with the MS/MS ion search tool in ProteinPilot software (AB SCIEX).

## Results

### Carbonylated proteins in DG after the ischemia-reperfusion

**Fig. 1:**
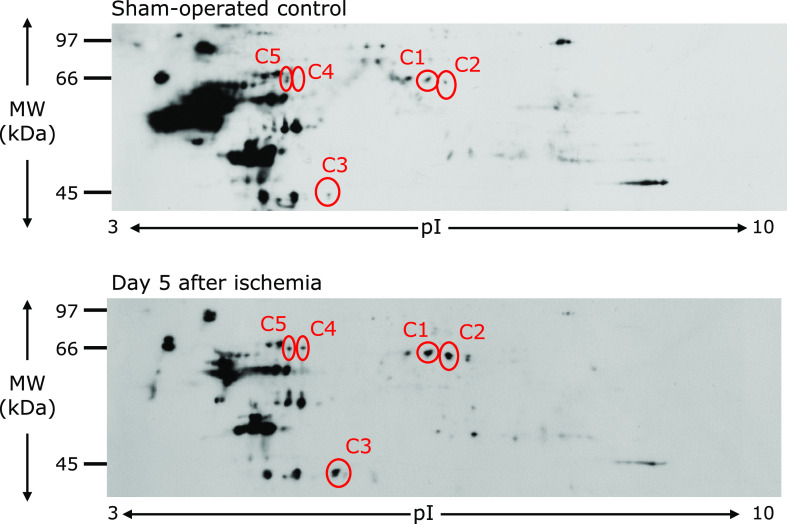
2D Oxyblot analysis of DG of the sham-operated control (top) and day 5 (bottom) after the ischemia-reperfusion insult. Proteins (100 µg) were treated with DNPH and separated by 2DE, and transferred onto a PVDF membrane. Membrane was incubated with anti-DNP primary antibody, followed by incubation with horseradish peroxidase-conjugated secondary antibody. The reaction was visualized with ECL. The images obtained were processed using PDQuest ver. 8.0 (Bio-Rad Laboratories Ltd.) to match spots and provide spot volumes. The spots were identified by PMF.

**Fig. 2:**
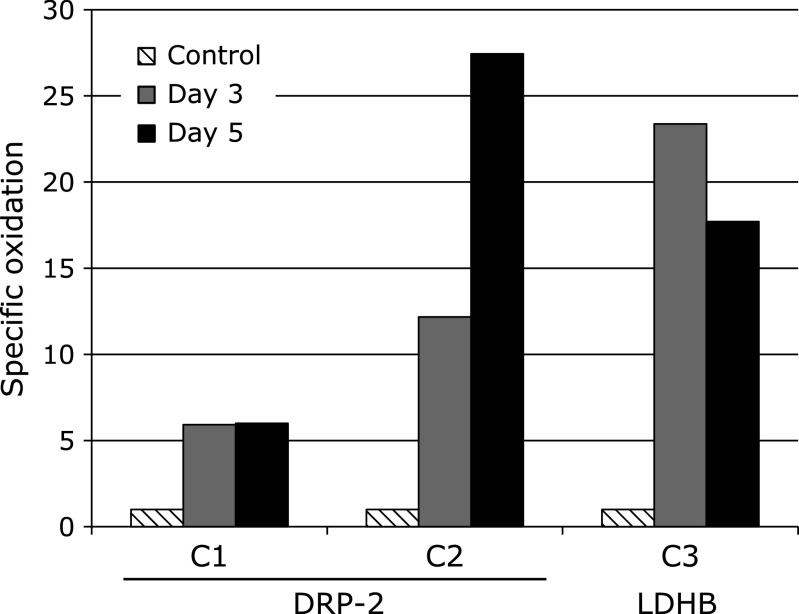
The time course of specific oxidation levels. Spot numbers are shown in Table 1 and Fig. 1. Data were presented by fold changes in the ischemia-reperfusion brains, being compared with the controls. The specific oxidation of each spot was calculated as relative carbonyl level per relative protein expression obtained from 2D DIGE.

To determine protein oxidation levels, protein carbonyls were studied by the 2D Oxyblot analysis of DNPH-treated samples, using an anti-DNP antibody. Figure 1 shows carbonylated proteins in the representative 2D Oxyblots from the DG of the control and day 5 after ischemia. A total of 192 carbonylated protein spots were detected in the DG. Among them, we focused on 4 spots which showed more than 5-fold increases in carbonylation at both days 3 and 5 after ischemia, compared to control. Among the 5 selected proteins, 3 spots could be identified by the mass spectrometry analysis followed by database matching. The spots of up-regulated carbonylated proteins were identified as dihydropyrimidinase related protein 2 (spot No. C1, C2) and l-lactate dehydrogenase B chain (spot No. C3). Information of these spots was described in Table 1, including the percentage of sequence coverage, protein name, theoretical molecular weight, theoretical pI-value, and functional role. The specific oxidation of each spot was calculated as relative carbonyl level per relative protein expression obtained from 2D DIGE. Figure 2 shows time course of the changes in the specific oxidation level.

### Protein expression profiles in DG after ischemia-reperfusion

**Fig. 3:**
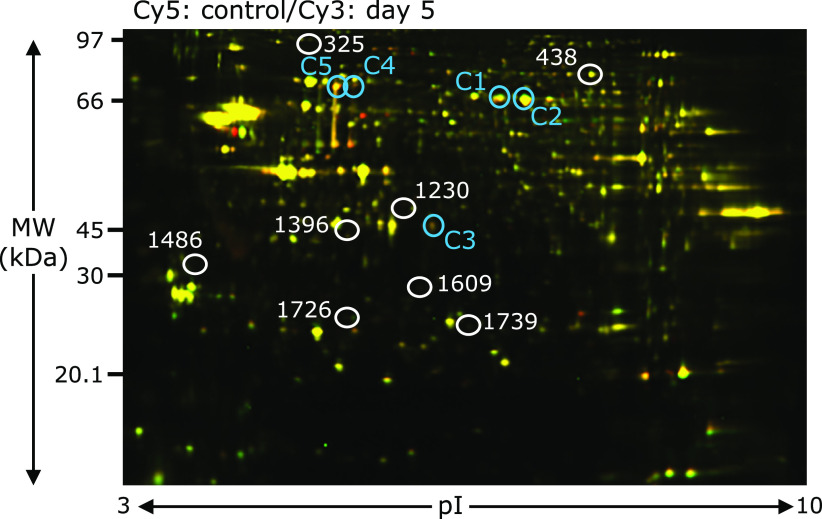
2D DIGE analyses of the control and day 5 postischemic DG. Proteins (25 µg) were labeled with Cy3 (red) or Cy5 (green) dyes, mixed and subjected to 2D DIGE analysis. Red spots indicate upregulated proteins on day 5 after the ischemic insult, while green spots indicate downregulated proteins. Yellow spots represent proteins that were unchanged. 8 differentially expressed spots (white circle) could be identified. The blue circle spots correspond to C1–C5 in Fig. 1.

We used 2D DIGE to investigate the protein expression profiles in DG at days 3, 5 and 7 after ischemia. Figure 3 shows the 2D DIGE image of DG lysates from the control and day 5 after ischemia. We detected spots on the 6 gels as determined by DeCyder Differerntial Analysis Software. A total of 2,372 protein spots were detected. Sixty-nine protein spots were significantly altered in the DG tissues of day 7 after ischemia, compared to the sham-operated control (*p* value <0.05 and relative protein expression level >|1.10|, *n* = 3/each group). Spots that were not detected in the Coomassie brilliant blue post-stained gel or poor quality spots were omitted. Finally, 8 differentially expressed spots could be identified by MALDI-TOF/TOF peptide mass fingerprinting (Table 2). The positions of the 8 spots (white circle) on the 2D gel are shown in Fig. 3.

The spots of up-regulated proteins were identified as NAD-dependent protein deacetylase sirtuin-2 (spot No. 1230), inorganic pyrophosphatase (spot No. 1396) and heat shock protein β-1 (spot No. 1739). The spots of down-regulated protein were identified as tropomyosin α-3 chain (spot No. 1486), heat shock cognate 71 kDa protein (spot No. 325), vesicle-fusing ATPase (spot No. 438), enoyl-CoA delta isomerase 1, mitochondrial (spot No. 1609) and PITH domain-containing protein 1 (spot No. 1726).

**Fig. 4:**
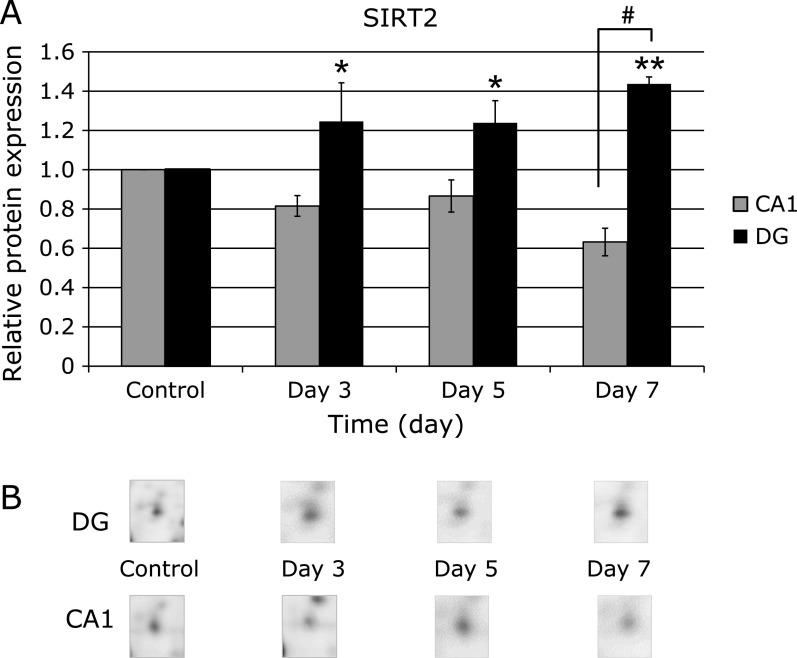
The time course of SIRT2 expression in DG and CA1 after the ischemia-reperfusion. (A) Graphical expression profile of SIRT2 spot. Data was presented by fold changes after the ischemia-reperfusion, being compared with the control; *p<0.05, **p<0.01 vs non-ischemic controls. ^#^ indicates a significant difference between DG and CA1 at 7 days after the ischemia-reperfusion by *t* test (*p*<0.01). (B) The representative SIRT2 spot image of 2D DIGE analysis.

Figure 4 shows the time course of the protein expression profiles of nicotinamide adenine dinueleotide (NAD)-dependent protein deacetylase sirtuin-2 (SIRT2) by comparing DG and CA1. SIRT2 was significantly increased in the DG at days 3, 5 and 7 after ischemia, but was decreased in the CA1.

## Discussion

### Alternations of carbonylated proteins in DG after ischemia-reperfusion

#### Heat shock 70 kDa protein 1 (Hsp70.1)

**Fig. 5:**
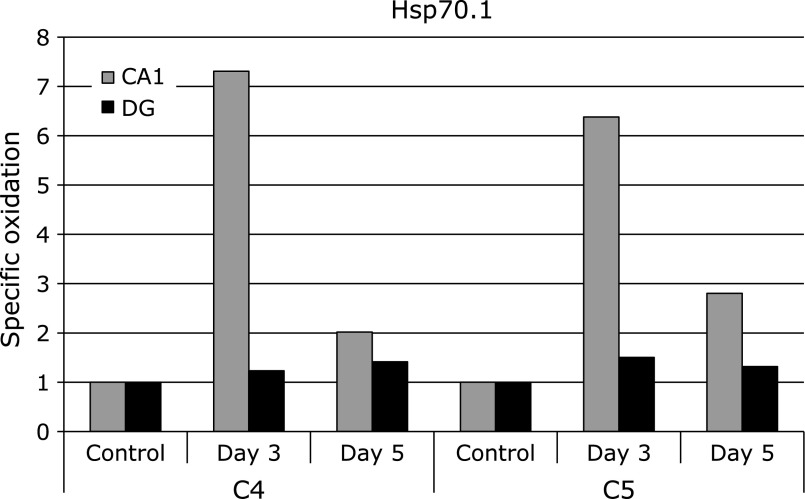
The time course of specific oxidation level of Hsp70.1 in DG and CA1 after the ischemia-reperfusion. Spot numbers of DG region were used in 2D Oxyblot analysis using DG. Data of DG were compared with our previously reported data of CA1.^(21)^

Figure 5 shows the time course of specific oxidation levels of Hsp70.1 in the CA1 and DG. Our previous study demonstrated that Hsp70.1 was remarkably oxidized in the CA1 at days 3 and 5 after ischemia. On the contrary, in the postischemic DG, carbonyl modification level of Hsp70.1 was increased only negligibly. Heat shock proteins (Hsps), being induced by stressful stimuli, are thought to support cellular integrity and viability. Hsp70.1 is a major protein of human Hsp70 family, and mainly functions as a chaperone enabling the cell to cope with harmful aggregations of denatured/damaged proteins during and after insults such as heat and ischemia.^(26,27)^ In addition, the expression of Hsp70.1 at the lysosomal membrane confers its stability against cell stresses.^(28–33)^ Previously, Sahara and Yamashima^(34)^ demonstrated that carbonylated Hsp70.1 in the CA1 after ischemia was cleaved by activated µ-calpain. Consequently, cleavage of Hsp70.1 leads to the membrane rupture with the resultant release of lysosomal protease cathepsins into the cytosol, which induced necrotic neuronal death in the CA1.^(33,35)^ Our results demonstrated the specific oxidation level of Hsp70.1 was extensively increased in the CA1 after ischemia, although it was increased only negligibly in the DG. Carbonyl modification of Hsp70.1 in the CA1 may lead to loss of the neuroprotection, whereas intact Hsp70.1 may play an important role for the protection of DG against oxidative stresses after ischemia.

#### Dihydropyrimidinase related protein 2 (DRP-2)

The specific oxidation levels of spot C1 and C2 were increased 5.99- and 27.43-fold, respectively, in the DG at day 5 after ischemia (Fig. 2). Both spots were identified as dihydropyrimidinase related protein 2 (DRP-2). DRP-2, also called ‘collapsing response mediator protein 2’ (CRMP-2) or ‘turned on after division-64 kD’ (TOAD-64), is a member of the CRMP/TOAD/Ulip/DRP family of cytosolic phosphoproteins. This protein family is involved in neuronal differentiation and axonal guidance, and found to be up-regulated after the focal brain ischemia.^(36)^ DRP-2 is found abundantly in the nervous system, especially during development, and have also been found in adult brain.

**Fig. 6:**
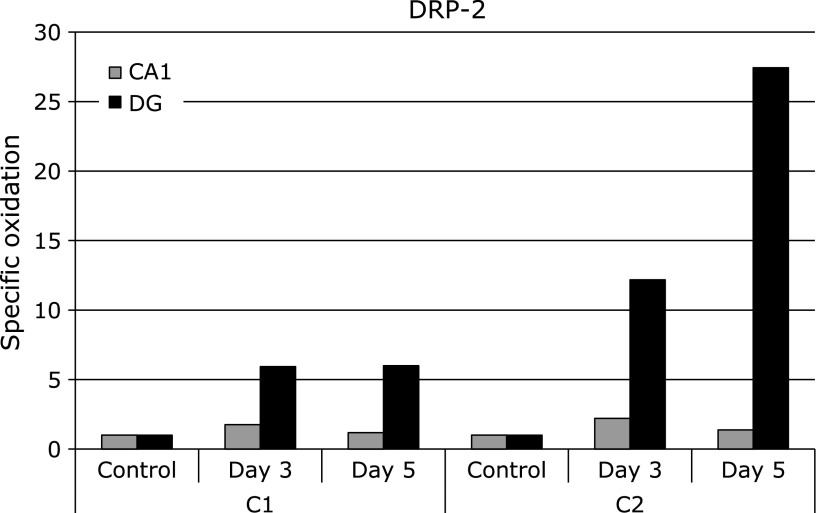
The time course of specific oxidation level of DRP-2 in DG and CA1 after the ischemia-reperfusion. Spot numbers of DG region were used in 2D Oxyblot analysis using DG. Data of DG were compared with our previously reported data of CA1.^(21)^

Our previous results showed that the specific oxidation level of DRP-2 in the monkey substantia nigra at day 5 after ischemia was increased more than 4-fold.^(37)^ Similarly, the specific oxidation level of DRP-2 in CA1 increased 2-fold on day 3 and 5 after ischemia. Furthermore, our results showed that the specific oxidation levels of spots C1 and C2 of DRP-2 in the DG at day 5 after ischemia increased more than 5-fold and 20-fold, compared to that of carbonylated DRP-2 in CA1, respectively (Fig. 6). It is considered that multifunctional protein DRP-2 is one of major determinants in the control of oxidative stress.^(38)^ Thus, a remarkably increase of specific oxidation level of DRP-2 in the postischemic DG may conceivably contribute to modulation of ischemic neuronal death. Fukui *et al.*^(39,40)^ reported that ROS-derived phospho-DRP-2 expression may be associated with the induction axonal degeneration. Therefore, investigation is needed to clarify the expression level of phospho-DRP-2 induced by the oxidative damage due to ischemia reperfusion.

#### l-Lactate dehydrogenase B chain (LDHB)

The specific oxidation level of spot C3 showed 17.7-fold increase in the DG at day 5 after ischemia (Fig. 2). Spot C3 was identified as l-lactate dehydrogenase B chain (LDHB). LDHB is terminal enzyme of anaerobic glycolysis, catalyze conversion of lactate into pyruvate.^(41)^ l-lactate dehydrogenase (LDH) is a tetrameric enzyme formed by combinations of two subunit (LDHA and LDHB), that catalyzes the reversible NAD-dependent interconversion of pyruvate to lactate.^(42)^ LDH activity in rat brains declined with the increasing age,^(43)^ and LDH activity reduced in hippocampus of mild cognitive impairment, which was correlated protein dysfunction and enzyme activity impairment.^(44)^ Poon *et al.*^(45)^ reported that LDH activity depression is caused by oxidative modification of enzyme. In addition, it is reported that LDH is carbonylated in the brain of SAMP8 mouse.^(45)^ These results suggested that carbonylated LDHB have some effect on the decline in learning ability and memory processes in hippocampus by oxidative stress.

### Comparison of protein expression between DG and CA1 after ischemia-reperfusion

Spot 1230 in Fig. 3 was identified as nicotinamide adenine dinucleotide (NAD)-dependent protein deacetylase sirtuin-2 (SIRT2). SIRT2 is widely accepted to be involved in ageing, energy production, and lifespan extension. SIRT2 expression is much higher in the brain than other organs, particularly in the cortex, striatum, hippocampus, and spinal cord.^(46)^ The present study first demonstrated that SIRT2 was significantly increased in the DG at days 3, 5 and 7 after ischemia, but was decreased in the CA1 (Fig. 4). It was recently reported that SIRT2 was upregulated in ischemic neurons *in vitro* and *in vivo*.^(47)^ Since SIRT2 activates an antioxidant enzyme, manganese superoxide dismutase (MnSOD),^(48)^ it can regulate the cellular response to oxidative stress by reducing ROS. As SIRT2 can also deacetylate forkhead box O1 (FOXO1), it may reveal the protective effects against oxidative stress.^(49)^ It is probable that upregulation of SIRT2 may contribute to the cell survival of the DG in response to the oxidative stress during the ischemia-reperfusion. Although further studies are needed to address a causal relationship between SIRT2 and cell death, SIRT2 should be a critical mediator of an oxidative stress response.

Other proteins such as inorganic pyrophosphatase and heat shock protein β-1 were significantly increased in the DG, but no significant changes were observed in the CA1. On the other hand, tropomyosin α-3 chain, heat shock cognate 71 kDa protein, vesicle-fusing ATPase, enoyl-CoA delta isomerase 1 (mitochondrial) and PITH domain-containing protein 1 were significantly decreased in the postischemic DG. These proteins also did not show a significant change in the CA1.

Inorganic pyrophosphatase catalyzes conversion of one molecule of pyrophosphate to two phosphate ions. It was reported that inorganic pyrophosphatase partially protected the cells from apoptosis.^(50)^ Cytosolic levels of inorganic pyrophosphatase have been shown to be increased in several proteomics studies dealing with cancer tissues such as lung and colon.^(51,52)^ Heat shock protein β-1 (also known as HSP-27) is a member of a small heat shock protein family and is involved in the regulation of apoptosis, protection of cells against oxidative stress.^(53)^ Interestingly, it is reported that heat shock protein β-1 is able to prevent α-synuclein aggregation in the culture of neurons overexpressing α-synuclein and thus increases neuron survival.^(54)^ Thus, the increase in expression levels of inorganic pyrophosphatase and heat shock protein β-1 may play a neuroprotective role in the DG after ischemia.

Tropomyosins are family of microfilament-associated structural proteins. It was reported that tropomyosin α-3 and -4 chain proteins participating in the cell motility were down-regulated in the vitamin C-induced apoptosis of human adenocarcinoma AGS cells.^(55)^ Tropomyosin stabilizes cytoskeleton actin filaments and as a result may participate in the morphological changes underlying hippocampal information processing.^(56)^ Heat shock cognate 71 kDa protein serves to protect neurons by recycling aggregated proteins and reducing their toxicity.^(57)^ Thus, downregulation of tropomyosin α-3 chain and heat shock cognate 71 kDa protein may be involved in the inhibition of apoptosis and neuroprotective in the DG after ischemia. Although enoyl-CoA delta isomerase 1 are involved in the basic mitochondrial function, the role of this protein is still unclear in the mechanism of cell survival in the DG after ischemia.

In conclusion, the DG is known to be extremely resistance to oxidative stress than the CA1 in hippocampus. In this study, the specific oxidation level of Hsp70.1 was extensively increased in the CA1 after ischemia, although it was not increased in the DG. In addition, the proteome analysis showed that the carbonyl levels of DRP-2 and LDHB were remarkably increased in the postischemic DG. Furthermore, protein expression level of SIRT2 was increased in DG, but it was decreased in CA1. We speculate that these proteins have the potential to be involved in the mechanism of resistance to oxidative stress in DG.

## Author Contributions

Contributors: Conceived and designed the study: SO and TY. Analyzed the data: SK, YK, YM, and ST-O. Wrote the paper: YM and SO. Paper modification: TY, HK and MM.

Ethics approval: The study was approved by the Animal Care and Ethics Committee of Kanazawa University (Approval No.: AP-132874).

## Figures and Tables

**Table 1:** Identification of carbonylated protein based on PMF

Spot number^a)^	% Cov^b)^	Protein name^c)^	MW^d)^	pI^e)^	Functional role
C1	28.3	Dihydropyrimidinase related protein 2 (DRP-2)	73,583	5.94	Axonal growth
C2	26.2				
C3	26.7	l-lactate dehydrogenase B chain (LDHB)	36,927	5.85	Metabolic enzyme

^a)^Spot number as indicated in Fig. 1. ^b)^Coverage of the matched peptides in relation to the full-length sequence. ^c)^Proteins identified by 2D-gel fingerprinting. ^d)^Theoretical molecular weight and ^e)^theoretical isoelectric point were searched using the ExPASy Compute pI/Mw tool in ExPASy website (http://www.expasy.org).

**Table 2:** Identification of differentially expressed protein spots in DG

Spot number^a)^	Protein name	% Cov^b)^	pI^c)^	MW^d)^	Relative protein expression level			
					Cont	Day 3	Day 5	Day 7
325	Heat shock cognate 71 kDa protein	13.2	5.37	70,898	1	0.92	0.88	0.79*
438	Vesicle-fusing ATPase	12.5	6.52	82,626	1	1	1	0.84*
1230	NAD-dependent protein deacetylase sirtuin-2	12.3	5.22	43,182	1	1.25*	1.23*	1.44*
1396	Inorganic pyrophosphatase	31.5	5.66	32,743	1	1.03	1.08	1.20*
1486	Tropomyosin α-3 chain	25.4	4.75	29,007	1	0.96	0.91	0.90*
1609	Enoyl-CoA delta isomerase 1, mitchondrial	9.3	8.80	32,816	1	0.96	0.88*	0.88*
1726	PITH domain-containing protein 1	24.2	5.47	24,178	1	0.91	0.88	0.79*
1739	Heat shock protein β-1	21.0	5.98	22,783	1	1.31	1.27*	1.27*

^a)^Spot number as indicated in Fig. 3. ^b)^Coverage of the matched peptides in relation to the full-length sequence. ^c)^Theoretical isoelectric point and ^d)^theoretical molecular weight were searched using the ExPASy Compute pI/Mw tool in ExPASy website (http://www.expasy.org). *p<0.05 compared to the control.

## Abbreviations

CA1: cornu *Ammonis* 1
2D DIGE: two-dimensional difference gel electrophoresis
2D Oxyblot: 2DE with immunochemical detection of protein carbonyls
2DE: two-dimensional gel electrophoresis
DG: dentate gyrus
DNP: 2,4-dinitrophenylhydrazone
DNPH: 2,4-dinitrophenylhydrazine
DRP-2: dihydropyrimidinase related protein 2
FOXO1: forkhead box O1
Hsp70.1: heat shock 70 kDa protein 1
PMF: peptide mass fingerprinting
ROS: reactive oxygen species
SIRT2: nicotinamide adenine dinucleotide (NAD)-dependent protein deacetylase sirtuin-2
